# Exploring Changes in Two Types of Self-Efficacy Following Participation in a Chronic Disease Self-Management Program

**DOI:** 10.3389/fpubh.2016.00196

**Published:** 2016-09-19

**Authors:** Kay Graham, Matthew Lee Smith, Jori N. Hall, Kerstin G. Emerson, Mark G. Wilson

**Affiliations:** ^1^School of Occupational Therapy, Brenau University, Gainesville, GA, USA; ^2^Department of Health Promotion and Behavior, College of Public Health, The University of Georgia, Athens, GA, USA; ^3^Department of Health Promotion and Community Health Sciences, Texas A&M Health Science Center, School of Public Health, College Station, TX, USA; ^4^Department of Lifelong Education, Administration, and Policy, College of Education, The University of Georgia, Athens, GA, USA; ^5^Department of Health Policy and Management, College of Public Health, The University of Georgia, Athens, GA, USA

**Keywords:** self-efficacy, chronic disease, self-management, fall prevention, chronic disease self-management program, principal component analysis

## Abstract

Chronic conditions and falls are related issues faced by many aging adults. Stanford’s Chronic Disease Self-Management Program (CDSMP) added brief fall-related content to the standardized 6-week workshop; however, no research had examined changes in Fall-related self-efficacy (SE) in response to CDSMP participation. This study explored relationships and changes in SE using the SE to manage chronic disease scale (SEMCD Scale) and the Fall Efficacy Scale (FallE Scale) in participants who successfully completed CDSMP workshops within a Southern state over a 10-month period. SE scale data were compared at baseline and post-intervention for 36 adults (mean age = 74.5, SD = ±9.64). Principal component analysis (PCA), using oblimin rotation was completed at baseline and post-intervention for the individual scales and then for analysis combining both scales as a single scale. Each scale loaded under a single component for the PCA at both baseline and post-intervention. When both scales were entered as single meta-scale, the meta-scale split along two factors with no double loading. SEMCD and FallE Scale scores were significantly correlated at baseline and post-intervention, at least *p* < 0.05. A significant proportion of participants improved their scores on the FallE Scale post-intervention (*p* = 0.038). The magnitude of the change was also significant only for the FallE Scale (*p* = 0.043). The SEMCD Scale scores did not change significantly. Study findings from the exploratory PCA and significant correlations indicated that the SEMCD Scale and the FallE Scale measured two distinct but related types of SE. Though the scale scores were correlated at baseline and post-intervention, only the FallE Scale scores significantly differed post-intervention. Given this relationship and CDSMP’s recent addition of a 10-min fall prevention segment, further exploration of CDSMP’s possible influence on Fall-related SE would provide useful understanding for health promotion in aging adults.

## Introduction

Although chronic disease has become an issue for over half of all adults in the U.S., older adults have an even higher rate for single and multiple chronic conditions (1). Older adults also face increasing risk of injury due to falls as they age (2). Risk of falls can be further affected by the direct effects of disease as well as indirect effects, such as weakness, limited engagement, and balance issues (3). Given the negative ramifications associated with chronic disease and falls among older adults, evidence-based programs (EBPs), especially those that focus on disease self-management and fall management and prevention, are key components of health promotion geared toward the older adult population (4). Stanford’s Chronic Disease Self-Management Program (CDSMP) is an EBP that uses self-efficacy (SE) and mastery experiences to develop skills and SE to manage chronic conditions (SEMCD) (5).

The Chronic Disease Self-Management Program promotes better health and better care through workshop content focused on exercise, diet, environmental safety, provider communication, and action planning/goal setting (5, 6). CDSMP and typical fall prevention programing share some general content, including the use of action plans, the importance of exercise, medication issues, effective communication, and focus on promoting SE (5, 7–10). The most recent version of CDSMP also added content to specifically address falls with a 10-min activity entitled “Preventing Falls and Improving Balance” (11). During this session, leaders review and brainstorm risks for falls and follow up with a review of ways to reduce fall risk (11). The intersecting issues of multiple conditions and fall risk may be at least partially addressed in an integrated manner through this addition of fall-related content (fall-specific and general) within a general self-management program, such as CDSMP.

In addition to overlapping program content, programing to promote managing conditions may also share some of the same target populations with fall prevention programing. Although both CDSMP and fall prevention programing are offered by agencies serving older adults; CDSMP workshops typically have younger participants with more conditions than many fall prevention program participants. For example, in the *National Study of CDSMP* (12), the average participant age was 65.4 years, while the average participant age in a large fall prevention study (13) was 77 years. In both types of programs, participants typically had at least one chronic condition (14, 15). For example, CDSMP participants self-reported an average of 3.0 conditions (15) and fall program participants self-reported an average of 1.64 conditions (14).

Self-efficacy, the perceived confidence in one’s ability to complete a task and exercise control (16), is often a key component of health promotion theories and programs (17). Both CDSMP and some fall prevention programs [e.g., A Matter of Balance/Volunteer Lay Leader Model (AMOB/VLL), Stepping On] utilize SE as a foundational program component to facilitate a sense of control, self-management and specific program outcomes (7, 18, 19).

Since SE is understood as task specific (16), short distinct SE scales have been developed depending on the type of program and the type and range of tasks associated with the interventions’ specific content and outcomes. More specifically, CDSMP as a program emphasizes a person’s SEMCD. The six-item SE to manage chronic disease scale (SEMCD Scale) is currently recommended for use by CDSMP researchers who noted that the SEMCD Scale was correlated for both baseline and post-intervention health indicators, such as health distress, illness intrusiveness, activity limitation, depression, and fatigue (18). Fall-related SE is incorporated into many fall prevention program research studies and has been measured using a variety of SE scales, such as the five-item fall management SE scale [Fall Efficacy Scale (FallE Scale)] (7, 20). Despite the overt relationship between chronic conditions and fall risk and the addition of fall-related content into CDSMP workshops, changes in Fall-related SE have yet to be assessed in conjunction with CDSMP participation.

**Figure 1:**
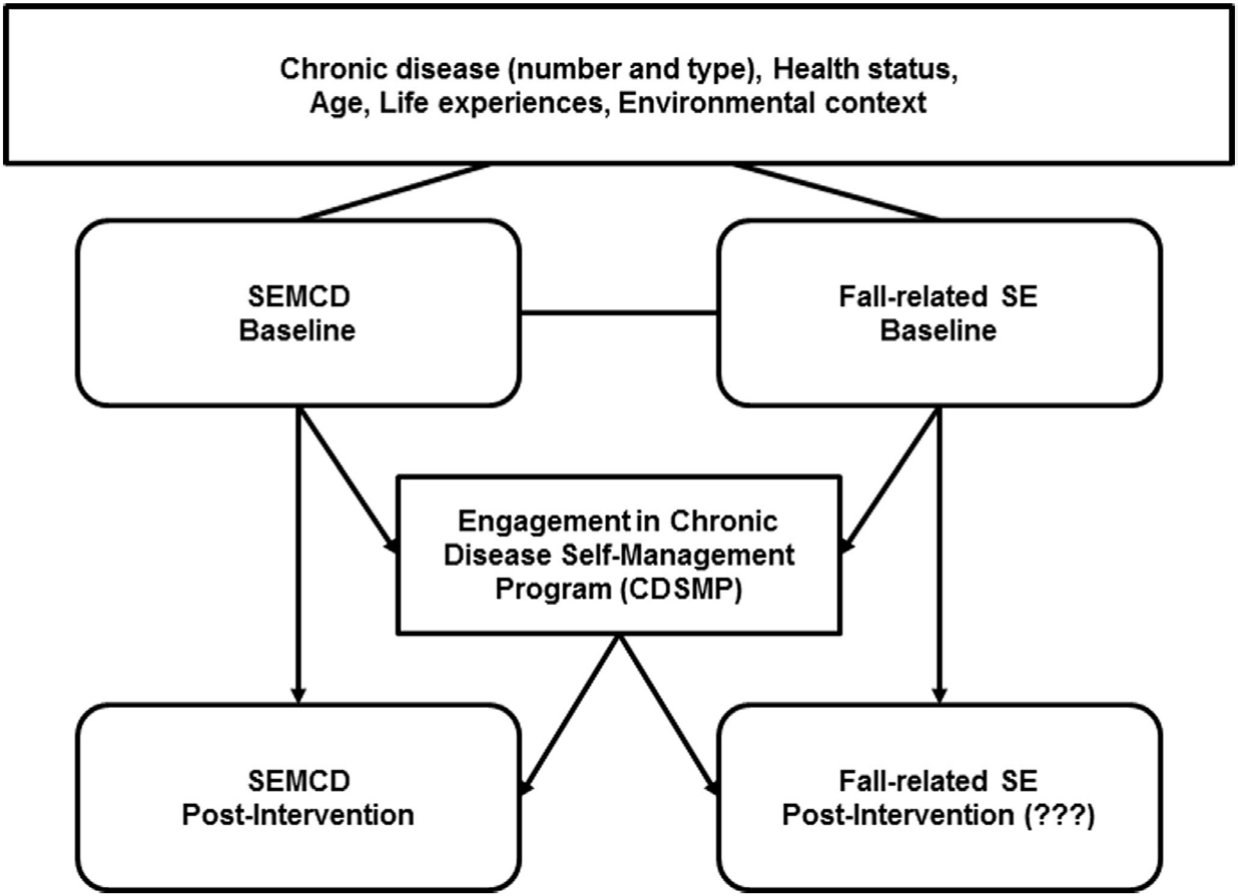
**CDSMP and types of self-efficacy**. Visual depiction of self-efficacy to manage chronic condition (SEMCD), Fall-related self-efficacy (Fall-related SE) and participation in Stanford’s Chronic Disease Self-Management Program (CDSMP). Arrows represent potential influences in self-efficacy (SE) at baseline and post-intervention (participation in CDSMP).

The intersection of aging, disease, and falls should be addressed in a broad approach that incorporates fall management into disease management (21) and acknowledges that “fall risk” often results from chronic issues (22). Fall prevention researchers have described complementary services, such as coordinated medical management of conditions, exercise programs, and home assessments to enhance fall management outcomes (22) and have advocated for a “no wrong door” approach to fall prevention (p. 270). The addition of fall-related content to CDSMP, as well as the potential intersections in terms of reaching older adults (who are seeking to manage conditions but also may be dealing with increased risk or concerns about falling possibly due to those conditions), make CDSMP a possible route to address fall prevention and management. It follows that natural next steps might explore possible changes to Fall-related SE following CDSMP participation. Figure 1 shows the theorized relationships between the participants’ personal characteristics as well as SE at baseline and post-intervention (SEMCD and Fall-related SE) as related to CDSMP participation.

This study offered an initial exploration at baseline and post-participation in CDSMP between two types of efficacies, SEMCD and Fall-related SE. The purposes of this study were to: (1) explore relationships between types of SE using SE scale scores for managing disease (SEMCD) and managing/preventing falls (Fall-related SE) and (2) assess changes in FallE Scale and SEMCD Scale scores after CDSMP participation. The following hypotheses were postulated: (1) improvements in SEMCD Scale scores would be observed following CDSMP participation; (2) improvements in FallE Scale scores would be observed following CDSMP participation; and (3) positive associations would be identified between SEMCD and FallE Scale scores.

## Materials and Methods

### Chronic Disease Self-Management Program and Recruitment

The University of Georgia Institutional Review Board approved this study as part of a larger mixed method study exploring the relationships between SEMCD and SE to manage and prevent falls (Fall-related SE) among older adults who successfully completed (attending 4+ of 6 sessions) CDSMP workshops. The standardized CDSMP promotes self-management skills, such as problem solving, decision making, using resources, interacting with providers, as well as setting goals to facilitate self-management of conditions (5). During the six 2.5-h workshop sessions, lay leaders use action planning, feedback, and social modeling to promote participant mastery and increase SEMCD (5). Content includes a brief section on fall prevention and balance as well as safe medication use, improving provider communications, the importance of activity/exercise, managing pain/fatigue, dealing with emotions/depression, positive thinking, diet, relaxation, and sleep.

Participants were recruited from CDSMP workshops being held within two main regional Area Agencies on Aging during a 10-month period. After the first 2 months, recruitment expanded to the entire state to maximize participant recruitment opportunities. Of the 19 classes scheduled in the two main regions, eight workshops were conducted and 11 workshops were canceled due to lack of registration or participation. The additional regional recruitment resulted in one out of two possible workshops yielding additional participants for research purposes.

**Figure 2:**
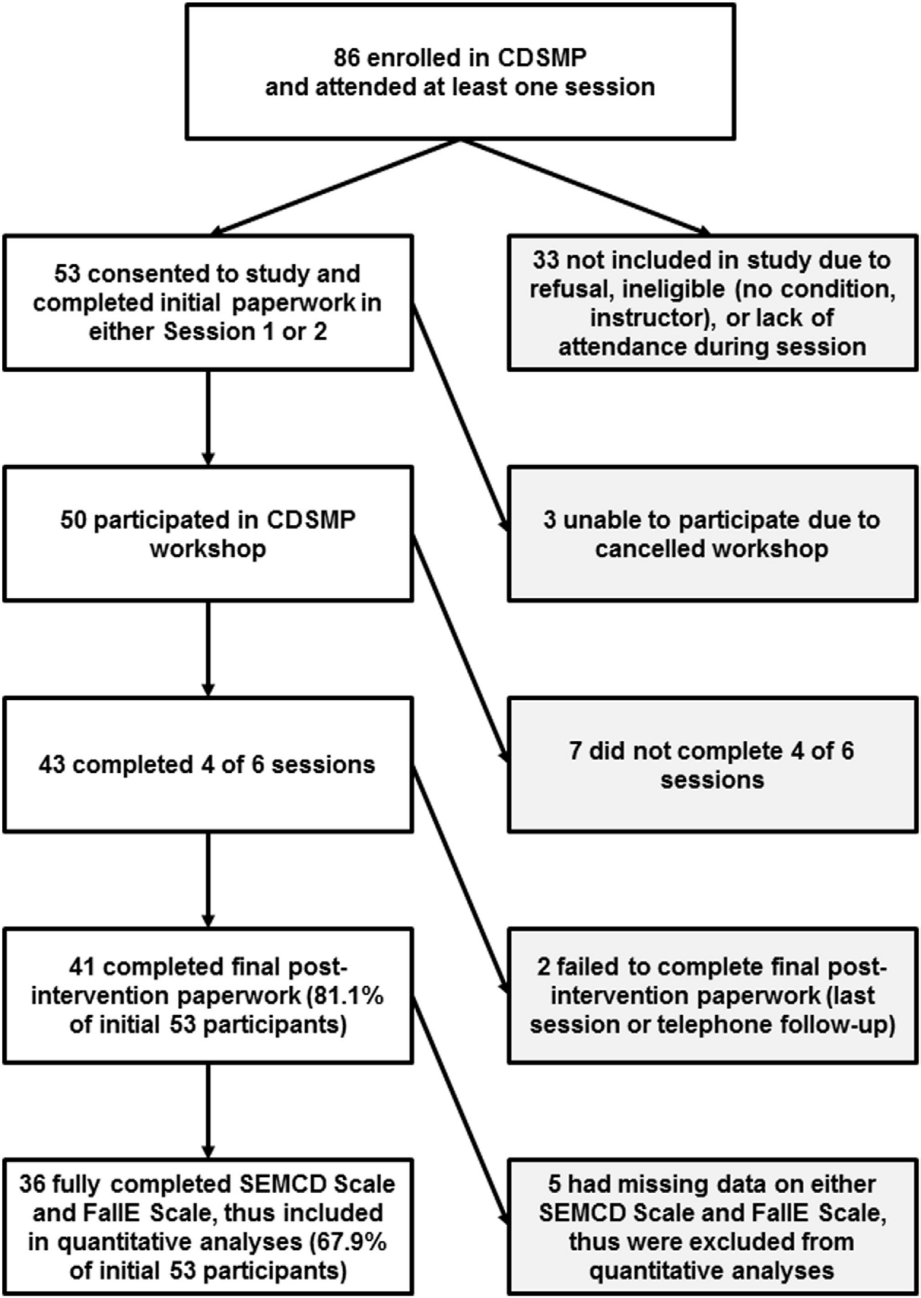
**Participant enrollment, convenience sample size, and selection criteria for use in analyses**. CDSMP, Chronic Disease Self-Management Program; SEMCD Scale, Self-Efficacy to Manage Chronic Disease Scale; FallE Scale, Fall Efficacy Scale.

Eligibility criteria were based in part on criteria used by the *National Study of CDSMP* (12), which required participants to have attended the first or second CDSMP workshop session, been diagnosed with a chronic disease, and consented to participate in study’s baseline and post-intervention data collection. To ensure receipt of intervention, only those who successfully completed the program (attending at least four of six sessions) were included in the final analyses. Of the total 86 CDSMP workshop participants, 53 consented to the study. Of those 53 who consented to the study, 43 completed the required 4+ sessions, and 36 of those 43 fully completed both the SEMCD Scale and the FallE Scale and were, therefore, used in analyses (see Figure 2 for specific breakdown of participant recruitment efforts). Across the rest of the state, one additional region’s class was recruited for the study. Another region agreed to assist with the study but was not included as the course was canceled. For three workshops (in other regions), either course site or course leaders deferred study participation.

### Measures

#### Demographics

To minimize participant burden, demographic information was retrieved from self-reported intake forms used for CDSMP workshops within the state. Permission to access this information was first obtained from the State Division of Aging Services and then only accessed with participant consent. Self-reported demographic information retrieved from this form included age, sex, race (American Indian, Asian/Asian-American, Black/African American, Hawaiian Native/Pacific Islander, White/Caucasian), ethnicity (Hispanic, non-Hispanic), chronic conditions (Alzheimer’s/Dementia, Osteoarthritis/Rheumatoid Arthritis, Breathing/Lung disease, Cancer, Chronic Pain, Depression/Anxiety, Diabetes, Heart Disease, High Cholesterol, Hypertension, Multiple Sclerosis, Osteoporosis, Stroke, Other, None), and education level (some elementary-high school, high school graduate or GED, some college or technical school, bachelor’s degree or higher).

#### SE Scales

Since this research explored relationships between SE to manage disease (SEMCD) and SE to manage/prevent falls (Fall-related SE) at baseline and following CDSMP participation, appropriate scales were needed to measure these distinct types of SE. The SEMCD Scale and the FallE Scale were chosen based on documented evidence of each scale’s good internal consistency and consistent loadings into single factors, respectively.

Consenting participants completed the initial baseline measures using the SEMCD Scale and the FallE Scale during session one or two of the CDSMP workshop. Post-intervention measures were collected from the same participants at the final session. In cases where the final session was missed, data were collected via phone follow-up. The researcher or lay leader provided limited support to those needing assistance to read and/or complete consent and scale forms. Details about each SE scale are provided below.

##### SEMCD Scale

Participants completed initial baseline and post-intervention responses for a 6-item modified version of the SEMCD Scale using a Likert scale with response choices ranging from 0 to 10 (0 = not at all confident to 10 = completely confident). Participants were asked: *How confident are you that you can*: (1) *Keep the fatigue caused by your disease from interfering with the things you want to do?* (2) *Keep the physical discomfort or pain of your disease from interfering with the things you want to do?* (3) *Keep the emotional distress caused by your disease from interfering with the things you want to do?* (4) *Keep any other symptoms or health problems you have from interfering with the things you want to do?* (5) *Do the different tasks and activities needed to manage your health condition so as to reduce your need to see a doctor?* (6) *Do things other than just taking medication to reduce how much your illness affects your everyday life?* Scores were reported as average scores. This 6-item format was developed and recommended by Stanford CDSMP researchers to measure SE for managing chronic conditions (23). Prior researchers have reported baseline SEMCD Scale mean scores ranging from 4.9 to 6.1 and 6-month post-intervention mean differences in scores ranging from 0.36 to 0.84 (18). Ritter and Lorig (18) noted the scale loaded on a single factor using principal component analysis (PCA) and had high internal consistency reliability coefficients (Cronbach’s alpha ranged from 0.88 to 0.95). They recommended the SEMCD Scale as a reliable scale for the measurement of SEMCD.

##### FallE Scale

There are many existing scales that examine Fall-related SE. For example, the Falls Efficacy Scale developed by Tinetti et al. (24), the Modified Falls Efficacy Scale (25), the Activities-Specific Balance Confidence Scale (26, 27), and the FallE Scale developed by Tennstedt et al. (7, 14, 20) have been used in a variety of studies to document older adults’ perceptions related to Fall-related SE. For this study, the 5-item Fall Efficacy Scale (FallE Scale) was selected to measure baseline and post-intervention Fall-related SE. For this current study, participants rated items using the 1–4 Likert scale (1 = not at all sure to 4 = very sure), regarding their *confidence to*: (1) *Find a way to get up if fall;* (2) *Find a way to reduce falls;* (3) *Protect self if fall;* (4) *Increase physical strength;* and (5) *Become more steady on feet*. Scores were summed as a total score using the recent scoring method used in translational study of an evidence-based fall management and prevention program (14).

The FallE Scale was developed by Tennstedt et al. (20) as a fall management scale. Since that time, the scale has been used to measure perceived ability (SE) to manage and/or prevent falls (Fall-related SE) in people attending the fall prevention program, AMOB/VLL (7). Reliability coefficients reported for this scale have ranged from 0.76 when initially developed (20) to 0.87 in recent translational studies (7, 14). Prior exploratory factor analysis established the scale as a continuous scale with potential total score ranging from 4 to 20 (7). Although this scale is not widely used outside of AMOB/VLL research, the FallE Scale was chosen as a measure of Fall-related SE because it focused specifically on confidence to manage and prevent falls, has had good internal consistency, and has factored as a single scale.

#### Statistical Analyses

To promote consistency of comparisons between participants, only participants with fully completed baseline and post-intervention scales were included in analyses for a final *n* = 36 out of the 53 consenting participants. SPSS was used for all statistical analyses. Demographics were reported as frequencies and percentages. Age and number of conditions were reported as means with standard deviation (SD). Average scores for the SEMCD Scale and total summed scores for FallE Scale were calculated and used for most analyses (i.e., PCA, correlations, and *t*-tests). Medians, proportions of participants with positive and negative score changes, and median differences were also calculated for Wilcoxon signed-rank tests.

Principal component analysis (using oblimin rotation with delta set at 0.0 and suppressing coefficients below 0.4) were completed for individual and combined scales at baseline and post-intervention. Oblimin rotation was chosen due to the correlations between the scales. A series of four principal component analyses were performed to assess the factor structure of the SEMCD Scale and the FallE Scale (i.e., SEMCD Scale baseline, SEMCD Scale post-intervention, FallE Scale baseline, and FallE Scale post-intervention). Internal consistency reliability coefficients were calculated using Cronbach’s alpha for each scale at both time points. For the final PCA, the SEMCD Scale and FallE Scale scores were entered into a single PCA as an initial exploratory technique to assess potential overlap of SE concepts at both time points (i.e., SEMCD/FallE Scale baseline and SEMCD/FallE Scale post-intervention).

Spread and distribution of data were checked using box plots, histograms, Q-Q plots, means, and analysis of median rankings. Sensitivity analyses with and without the outliers were also completed to assess possible changes in outcomes due to outliers. Pearson correlations were performed to identify the strength and direction of hypothesized relationships between the two types of SE at baseline and post-intervention. Due to data being evenly but not normally distributed, Wilcoxon signed-rank tests were used to analyze proportions of participants who changed or stayed the same. Paired sample, two-tailed *t*-tests were also performed for each question and for total scales (average score for SEMCD Scale and total summed score for FallE Scale).

## Results

Among the participating course locations, there were 86 possible participants in the CDSMP workshops, of which 63 (73.3%) successfully completed the course (attended 4+ of 6 sessions). Fifty-three out of a possible 86 agreed to participate in this study. Of those 53, 43 completed the required 4+ of 6 sessions. Although 41 of the 43 completers completed both scales at both time points, only 36 of the 41 had answered all items for both scales at both time points. Therefore, the final data analyses used the 36 participants who had attended at least 4 CDSMP workshop sessions and also had fully complete scale data at both time points.

Of those 36 participants, the mean age was 72.79 with 7 (20.5%) participants below the age of 65 years (see Table 1 for sample descriptives). Most of participants were female (77.8%). Most classified themselves as White (75%) and/or African American (25%). Of those reporting education level, 6% had some elementary or high school education, 30.3% reported having graduated from high school, 33.33% reported some college or technical school, and 30.3% reported having bachelor’s degree or higher. The leading five conditions reported by participants included hypertension (45.7%), high cholesterol (42.9), arthritis (42.9%), diabetes (37.1%), and breathing/lung issues (31.4%). Those participants used in the final analyses reported an average of 3.63 conditions (SD ± 2.5) and attended an average of 5.31 sessions (SD ± 0.749) (see Table 1 for demographics from consenting participants).

**Table 1:** **Participant baseline characteristics**.

	All consenting to study	Used in analysis	Consented but not included in analysis

	Mean (±SD)	Mean (±SD)	Mean (±SD)
Age in years	74.45 (±9.64)	72.19 (±8.19)	76.27 (±11.74)
Number of conditions reported per person	3.95 (±2.43)	3.63 (±2.5)	4.36 (±2.0)
Number of sessions attended per person	4.65 (±1.55)	5.31 (±0.749)	3.31 (±1.89)

***N* varies with # responses**	***N* (%)**	***N* (%)**	***N* (%)**

Age frequencies	*N* = 42	*N* = 34	*N* = 11
<65	8 (19.0)	7 (20.5)	2 (18)
≥65	34 (81.0)	27 (79.4)	9 (82)
Gender	*N* = 52	*N* = 36	*N* = 16
Female	41 (78.8)	28 (77.8)	13 (81.3)
Male	11 (21.1)	8 (22.2)	3 (18.8)
Race/ethnicity (more than one possible)	*N* = 44	*N* = 35	*N* = 12
Caucasian/White	33 (75.0)	27 (75)	8 (66.7)
African American	13 (29.5)	9 (25)	5 (41.7)
American Indian	4 (7.5)	4 (8.3)	1 (8.3)
Asian	2 (4.5)	2 (2.8)	1 (8.3)
Hispanic	0 (0)	0 (0)	0 (0)
Education	*N* = 41	*N* = 33	*N* = 11
Some elementary to high school	4 (9.8)	2 (6.06)	2 (18.2)
High school graduate or GED	10 (24.4)	10 (30.30)	0
Some college or technical school	17 (41.5)	11 (33.33)	7 (63.6)
Bachelor’s degree or higher	10 (24.4)	10 (30.30)	2 (18.2)
Chronic conditions	*N* = 43	*N* = 35	*N* = 11
Alzheimer’s/dementia	1 (2.3)	1 (2.9)	0 (0.0)
Osteoarthritis/rheumatoid arthritis	22 (51.2)	15 (42.9)	8 (72.7)
Breathing/lung	12 (27.9)	11 (31.4)	1 (9.1)
Cancer	3 (7.0)	3 (8.6)	0 (0.0)
Chronic pain	11 (25.6)	7 (20)	5 (45.5)
Depression/anxiety	10 (23.3)	10 (28.5)	1 (9.1)
Diabetes	19 (44.2)	13 (37.1)	7 (63.6)
Heart disease	8 (18.6)	6 (17.1)	2 (18.2)
High cholesterol	20 (46.5)	15 (42.9)	6 (54.5)
Hypertension	21 (48.8)	16 (45.7)	7 (63.6)
Osteoporosis	6 (14)	5 (14.3)	1 (9.1)
Stroke	4 (9.3)	2 (5.7)	3 (27.3)
Other conditions	19 (44.2)	14 (38.9)	4 (36.4)

*Total possible N = 16 and includes 7 who did not complete 4+ of 6 sessions; 6 with incomplete or missing scales; 3 who consented and course was cancelled*.

### Data Distribution

Listwise use of data (participants with fully complete scale scores) facilitated consistent comparisons across the results. Results were essentially unchanged before and following sensitivity checks for outliers. Based on boxplot visuals, outliers were generally evenly distributed around the mean for both scales (exception for FallE Scale items: “Steady on feet” and “Increase strength”). Outliers were retained based on these overall results. The scale scores and differences did not generally have a normal distribution curve as assessed with Shapiro–Wilkes tests; however, data did have even distribution around the means, close orientation of medians, and sample size >30 which permitted an assumption of approximately normal distributions of the sampling distributions (28) needed to run correlations, Wilcoxon signed-rank tests, and paired *t*-tests.

### Principal Component Analysis and Reliability

Table 2 provides the factor loadings and communalities for items at baseline and post-intervention for the individual SEMCD Scale and the FallE Scale. Though sample size was small, data met criteria for good sampling adequacy (>0.8) using Kaiser–Meyer–Olkin test for both scales at baseline and post time points as well as individual item adequacy on anti-image correlation with values above >0.5 minimum. PCA of each individual scale loaded as expected based on prior scale reporting (7, 18) with one factor only at baseline and post-intervention for each scale. Placement of both scales together into the PCA using exploratory oblique rotations loaded into two components delineated along the two scales with no double loadings above 0.37 for either baseline or post time points. Factor 1-*conditions* accounted for 56% of the variance at baseline or post. Factor 2-*falls* accounted for 13% of variance at baseline and 14.83 of variance post-participation. Refer to Table 3 for more information. Reliability scores for SEMCD Scale were 0.94 and 0.95 for baseline and post-intervention scores, respectively (reported in Table 2). For the FallE Scale, scores were 0.81 and 0.79 that are considered acceptable alpha levels (28).

**Table 2:** **Principal component analysis of SEMCD Scale and FallE Scale**.

	Factor loading	Communality estimates	Factor loading	Communality estimates
**SEMCD Scale Items (confidence to…)**	**Baseline (α = 0.935)**		**Post-test (α = 0.950)**	
1. Keep fatigue from interfering with the things you want to do?	**0.93**	0.87	**0.94**	0.88
2. Keep pain/physical discomfort form interfering with the things you want to do?	**0.90**	0.81	**0.86**	0.74
3. Keep emotional distress from interfering with the things you want to do?	**0.93**	0.87	**0.83**	0.69
4. Keep other symptoms from interfering with the things you want to do?	**0.94**	0.89	**0.95**	0.91
5. Do the different task and activities needed to manage so as to reduce your need to see a doctor?	**0.64**	0.40	**0.87**	0.75
6. Do things other than just taking medications to reduce how much your illness affects your everyday life?	**0.91**	0.82	**0.91**	0.83
Eigenvalues	4.66		4.8	
% variance	77.59		80.00	
**FallE Scale Items (how sure are you that you can…)**	**Baseline (α = 0.810)**		**Post-test (α = 0.790)**	
1. Find a way to get up if you fall	**0.79**	0.62	**0.41**	0.17
2. Find a way to reduce falls	**0.69**	0.47	**0.81**	0.66
3. Protect yourself if you fall	**0.82**	0.68	**0.84**	0.70
4. Increase your physical strength	**0.66**	0.44	**0.88**	0.78
5. Become more steady on your feet	**0.80**	0.64	**0.78**	0.60
Eigenvalues	2.85		2.92	
% variance	56.89		58.31	

*Note: Items that loaded on a factor with a value of 0.4 or higher are presented in bold*.

*SEMCD Scale, Self-Efficacy to Manage Chronic Conditions Scale; FallE Scale, Fall Efficacy Scale*.

**Table 3:** **Principal component analysis of combined SEMCD Scale and FallE Scale items**.

	Rotated component (factor) loadings			
	Baseline		Post-test	
	Factor 1-conditions	Factor 2-falls	Factor 1-conditions	Factor 2-falls
**SEMCD Scale Items (confidence to…)**				
1. Keep fatigue from interfering with the things you want to do?	**0.85**	0.14	**0.92**	0.02
2. Keep pain/physical discomfort form interfering with the things you want to do?	**0.75**	0.25	**0.81**	0.11
3. Keep emotional distress from interfering with the things you want to do?	**0.84**	0.18	**0.82**	0.01
4. Keep other symptoms from interfering with the things you want to do?	**0.89**	0.10	**0.97**	−0.05
5. Do the different task and activities needed to manage so as to reduce your need to see a doctor?	**0.80**	−0.25	**0.86**	0.03
6. Do things other than just taking medications to reduce how much your illness affects your everyday life?	**0.84**	0.13	**0.92**	−0.05
**FallE Scale Items (how sure are you that you can…)**				
1. Find a way to get up if you fall	0.07	**0.74**	−0.22	**0.63**
2. Find a way to reduce falls	0.18	**0.59**	0.37	**0.59**
3. Protect yourself if you fall	0.14	**0.74**	0.14	**0.78**
4. Increase your physical strength	−0.18	**0.80**	0.27	**0.73**
5. Become more steady on your feet	0.28	**0.63**	0.13	**0.72**
Eigenvalues	6.21	1.44	6.18	1.63
% of variance	56.41	13.06	56.19	14.83

*Note: Items that loaded on a factor with a value of 0.4 or higher are presented in bold*.

### Correlations

The linear nature between SEMCD and FallE Scale scores was established via scatterplots. Correlations using Pearson’s and Spearman’s correlation coefficients were completed between the scales at each time point (i.e., baseline to baseline, post-intervention to post-intervention, and baseline to post-intervention) (see Table 4 for summarized coefficients). Both within-scale correlations for baseline and post-intervention time points were significant at *p* < 0.001 as were between scale correlations for baseline SEMCD Scale and baseline FallE Scale, post-intervention SEMCD and FallE Scales, and baseline SEMCD Scale and post FallE Scale. Post SEMCD Scale score and baseline FallE Scale score were significant at *p* = 0.049 (see Table 4 for specifics regarding Pearson correlations). Spearman correlations using ranked scores were also performed to fully address non-normal distribution. Similar significant levels were obtained. Spearman coefficients are available in appendices of associated dissertation (29).

**Table 4:** **Correlations for SEMCD Scale and FallE Scale at Baseline and Post-intervention in CDSMP Workshop (*N* = 36)**.

	Pearson’s *r*	
	*r*	*P*
Baseline SEMCD Scale and post SEMCD Scale	0.57***	<0.001
Baseline SEMCD Scale and baseline FallE Scale	0.61***	<0.001
Baseline SEMCD Scale and post FallE Scale	0.69***	<0.001
Post SEMCD Scale and baseline FallE Scale	0.33*	0.049
Post SEMCD Scale and post FallE Scale	0.52**	0.001
Baseline FallE Scale and post FallE Scale	0.74***	<0.001

Two-tailed significance: *P < 0.05, **P < 0.01, ***P < 0.001.

### Differences between Baseline and Post-Intervention

#### Wilcoxon Signed-Rank Test

A non-parametric Wilcoxon signed-rank test was used assess differences between baseline and post-intervention for the 36 participants with fully completed scale data (see Table 5 for item specifics using the Wilcoxon signed-rank tests for individual scale items as well as total scale scores). For SEMCD Scale, 18 participants had a positive difference in post scores overall (improved SEMCD Scale score from baseline to post-intervention), 6 participants had negative differences (SEMCD Scale score decreased from baseline to post-intervention), and 12 participants kept the same sum at baseline and post-intervention; however, despite more participants with positive changes, a Wilcoxon signed-rank test failed to demonstrate a significant median increase in post-participation scores as compared to baseline SEMCD scores following participation in CDSMP (*z* = 0.257, *p* = 0.797). The median of the differences for SEMCD was 0.83.

**Table 5:** **Wilcoxon signed-rank tests for SEMCD Scale and FallE Scale scores post-intervention minus baseline in CDSMP workshop (*N* = 36)**.

	Positive^a^	Neutral^b^	Negative^c^	Test	*P*
**SEMCD Scale Items (confidence to…)**					
1. Keep fatigue from interfering with the things you want to do?	10	18	8	−0.286	0.775
2. Keep pain/physical discomfort form interfering with the things you want to do?	12	13	11	0.214	0.830
3. Keep emotional distress from interfering with the things you want to do?	13	14	9	0.573	0.567
4. Keep other symptoms from interfering with the things you want to do?	12	15	9	0.317	0.751
5. Do the different task and activities needed to manage so as to reduce your need to see a doctor?	12	11	13	−0.780	0.435
6. Do things other than just taking medications to reduce how much your illness affects your everyday life?	10	16	10	−0.659	0.510
SEMCD Scale (possible scores from 0 to 10)	18	12	6	0.257	0.797
**FallSE Scale Items (how sure are you that you can…)**					
1. Find a way to get up if you fall	13	21	2	2.387	0.017
2. Find a way to reduce falls	15	17	4	2.599	0.009
3. Protect yourself if you fall	12	16	8	0.778	0.437
4. Increase your physical strength	9	19	8	−0.232	0.817
5. Become more steady on your feet	8	19	9	0.25	0.802
FallSE Scale (possible scores from 1 to 4)	19	8	9	2.073	0.038

*Note: The SEMCD Scale for this research was modified from a 1–10 possible score range to a 0–10 possible score range*.

*^a^Positive difference, improvement in scale scores from baseline to post-intervention*.

*^b^Neutral, no change from baseline to post-intervention*.

*^c^Negative difference, decrease in scale scores from baseline to post-intervention*.

For the total scale summed scores on the FallE Scale, 19 participants had a positive difference in post scores (improved FallE Scale score from baseline to post), 9 participants had a negative difference (lower FallE Scale score at post-intervention as compared to baseline), and 8 participants kept same sum baseline and post-intervention. The median of the differences for the FallE Scale scores was 1.0. The Wilcoxon signed-rank test produced a statistically significant median increase in post-intervention scores as compared to baseline Fall SE scores following participation in CDSMP (z = 2.073, *p* = 0.038). This was a small to medium effect size (*r* = 0.244).

#### Paired *t*-Tests

Table 6 lists mean and SDs for individual questions as well as total scale scores. Mean SEMCD Scale score and SD were 7.46 (±1.74) at baseline and 7.41(±1.86) at post-intervention. No mean differences were significant for SEMCD Scale individual items or for the full scale. For the FallE Scale, mean baseline was 13.86 (±1.68) and 14.69 (±3.26) at post-intervention. The FallE Scale mean total score difference had a positive increase following the CDSMP course from baseline to post-intervention at 0.83 (95% CI, 0.0265–1.640) with a medium effect size (*r* = 0.327). These mean differences were reflected in the statistically significant increase in SE as measured on the FallE Scale from baseline to post-participation in the CDSMP course [*t* (35) = 2.097, *p* = 0.043]. Two individual questions on the FallE Scale also had substantial improvements: “find a way to get up if you fall” [*t* (35) = 2.646, *p* = 0.012] and “find a way to reduce falls” [*t* (35) = 2.786, *p* = 0.009].

**Table 6:** **Paired sample *t*-tests for SEMCD Scale and FallE Scale scores post-intervention minus baseline in CDSMP workshop, *N* = 36**.

			Paired Differences					
	Baseline, mean (±SD)	Post, mean (±SD)	Mean diff (±SD)	*t* (df35)	SE	*p*	95% CI	
							Lower	Upper
**SEMCD Scale Items (confidence to…)**								
1. Keep fatigue from interfering with the things you want to do?	7.19 (±2.08)	7.08 (±2.14)	−0.11 (±1.85)	−0.361	0.31	0.72	−0.74	0.51
2. Keep pain/physical discomfort form interfering with the things you want to do?	7.22 (±2.22)	7.25 (±2.06)	0.03 (±2.30)	0.072	0.39	0.94	−0.75	0.81
3. Keep emotional distress from interfering with the things you want to do?	7.31 (±1.97)	7.50 (±1.99)	0.19 (±2.15)	0.543	0.36	0.59	−0.53	0.92
4. Keep other symptoms from interfering with the things you want to do?	7.36 (±1.96)	7.42 (±2.10)	0.06 (±2.30)	0.145	0.38	0.89	−0.72	0.84
5. Do the different task and activities needed to manage so as to reduce your need to see a doctor?	7.67 (±2.14)	7.42 (±2.06)	−0.25(±1.71)	−0.875	0.29	0.39	−0.83	0.33
6. Do things other than just taking medications to reduce how much your illness affects your everyday life?	8.03 (±1.63)	7.81 (±2.10)	−0.22 (±2.00)	−0.666	0.33	0.51	−0.90	0.46
SEMCD Scale (possible scores from 0 to 10)	7.46 (±1.74)	7.41 (±1.86)	−0.051 (±1.68)	−0.182	0.28	0.86	−0.62	0.52
**FallE Scale Items (how sure are you that you can…)**								
1. Find a way to get up if you fall	2.58 (±0.97)	2.92 (1.02)	0.33(±0.76)	2.646	0.13	0.012	0.08	0.59
2. Find a way to reduce falls	2.92 (±0.91)	3.31 (±0.75)	0.39 (±0.84)	2.786	0.14	0.009	0.11	0.67
3. Protect yourself if you fall	2.58 (±0.94)	2.69 (±0.89)	0.11 (±0.85)	0.780	0.14	0.441	−0.18	0.40
4. Increase your physical strength	3.00 (±0.76)	2.97 (±0.81)	−0.03 (±0.81)	−0.206	0.14	0.838	−0.30	0.25
5. Become more steady on your feet	2.78 (±0.93)	2.81 (±0.92)	0.03 (±0.91)	0.183	0.15	0.856	0.28	0.34
FallE Scale Sum Mean (possible scores from 4 to 20)	13.86 (1.68)	14.69 (3.26)	0.83 (2.38)	2.097	0.40	0.043	0.03	1.64

## Discussion

This study explored baseline and post-intervention relationships between the SEMCD and the SE to manage and prevent falls (Fall-related SE) for successful completers (4+ sessions) of CDSMP workshops. The significant changes in Fall-related SE supported the initial research purpose to explore possible changes to Fall-related SE due to the possible intersections between falls and chronic conditions as well as the addition of fall-related content into CDSMP. Lack of significant changes in SEMCD was somewhat unexpected because SEMCD (as measured by the SEMCD Scale) has been shown to have low to moderate effect sizes following CDSMP (9, 18).

The significant result for Fall-related SE but not SEMCD might be explained due to differences in participants recruited for this study as compared to participants in previous CDSMP-related studies. This study’s participants were different from recently published research on CDSMP in relation to age, number of conditions, and SEMCD mean. For example, the mean age of the participants included in this analysis was 72.9, 7.5 years higher than the 65.4 mean age reported from the *National Study of CDSMP* (12). It is of note that participants in this study were younger than the mean age (77 years) identified in recent fall prevention research using the FallE Scale (13).

Participants in the current study also reported a higher number of conditions (3.63) as compared to the average 3.0 conditions reported by participants in the *National Study of CDSMP* (12). The higher number of conditions could be a reflection of the older mean age of participants in this study since the number of chronic conditions increases with age (1); however, this age explanation would not be supported by recent data from the *National Study of CDSMP* where younger participants (age <65 years) had higher numbers of conditions and larger effect sizes on outcomes than the ≥65 group (15).

In addition, in this study, there was a possible ceiling effect in the sample that could have limited the post-intervention SEMCD score changes since the SEMCD Scale mean was already high at 7.46 (SD ± 1.71) out of 10 at baseline. This mean is higher than reported mean SEMCD ranges of 4.9 to 6.1 in other CDSMP research (18). High baseline scores indicate that these participants were already confident about their ability to manage their conditions when they entered the program despite their older age and multiple conditions.

In summary, the participants recruited for the current study were older, reported having more conditions, and started the workshop having more confidence to manage their conditions than other recent CDSMP studies. These differences from “typical” CDSMP participants may help explain why changes were noted in Fall-related SE but not SEMCD following CDSMP participation. Perhaps these confident, older CDSMP participants who have chosen to actively manage their condition(s) through participating in the CDSMP workshop also experience an unexpected boost to Fall-related SE. The small sample size in this research prohibited further exploration of differences, such as by age or conditions. Future research could explore if age or number of conditions would be associated with greater changes in Fall-related SE following CDSMP participation since fall risk has been shown to increase with the number of conditions (3).

The PCA factoring as single components for each scale at both time points supported prior research that each scale represented a distinct construct or type of efficacy (7, 18). The two-component PCA division along the scale items (with no double loading >0.37) when both scales were loaded at once further suggests distinct types of efficacy as measured by the two scales. The distinct types of self-efficacies represented by the SEMCD Scale and FallE Scale support the unique tasks and natures of different types of SE described by Bandura (16). The large and significant positive correlations and relationships between the scale scores justified the choice of oblimin type of rotation for PCA. These relationships were noted at either time point which suggests that the scales (SEMCD Scale and FallE Scale) might be related measures of different types of SE regardless of CDSMP workshop participation. Additional research could further explore participant understanding of the relationship between efficacies to manage falls (Fall-related SE) and to manage conditions (SEMCD).

As mentioned previously, changes to Fall-related SE following CDSMP participation had not been researched although significant improvements in Fall-related SE following participation in an evidence-based fall management and prevention programs had been well documented (7, 14, 19, 20). The positive proportional and magnitude changes noted in this research for the FallE Scale in participants following CDSMP should be further explored to determine if differences exist in other samples of CDSMP participants. Most surprising, one of the FallE Scale items that differed significantly (Getting up) was not specifically addressed anywhere within the CDSMP structured curriculum. While such changes in Fall-related SE are commonly measured and expected for older adult participants in fall prevention and management programs (7, 19), these significant changes occurred following participation in a general self-management program (CDSMP) that had only limited direct instruction about fall prevention.

Although SE is specific to the task at hand (in this case managing conditions or managing falls), SE generalizes when mastery experiences have similar subskills (16). Skills, such as problem solving are addressed in both types of programs. Successful problem solving during the workshop might have transferred to Fall-related SE. The participants could believe they were then also capable to manage falls. Generalization of overarching *self-regulatory skills* (in this case perhaps general self-management skills) could have also affected more specific perceptions of Fall-related SE (16). Future research with older adults could explore how CDSMP participants view Fall-related SE following workshop participation in order to gain additional understanding regarding changes to Fall-related SE within CDSMP. Researchers could also examine whether common skillsets promote generalization of similar subskills from CDSMP to fall-related content as well as explore possible overarching self-management influence.

### Limitations

Study limitations arose from the type of data collected and the limited sample size. This study did not collect whether participants had concurrent or prior participation in fall prevention programing, which could have influenced baseline and post-intervention FallE Scale scores (and associated changes). Gathering this information is recommended for future research. Additionally, asking participants whether or not they had previously participated in one or more EBP prior to attending CDSMP would have better contextualized their SE levels at baseline and SE improvements over time. Both scales relied on self-report data that may have resulted in recall bias or have been influenced by other events or even programs co-occurring during the intervention.

This current study’s small sample size (*n* = 36) limited the power to detect change as significant as well as increased susceptibility to skewed results. Power was sufficient for correlations at 0.94; however, the study was underpowered for the Wilcoxon signed-rank test (power at 0.48, for example, for sum difference in FallE Scale) and for the *t*-tests (power for FallE Scale sum difference at 0.53). Since data collection did not reach the numbers needed for statistical power, no analyses by group could be performed. However, this study’s results were resilient following sensitivity testing regardless whether outliers were excluded or included. Results also remained consistent whether parametric or non-parametric testing was utilized in response to non-normal data. The PCA results also were consistent with findings from other studies in terms of the individual scales and reliability (18). It is recommended that future studies replicate this study’s methods and analyses with larger samples to further assess changes in Fall-related SE as a result of CDSMP participation.

Additional limitations include lack of random assignment or comparison group (those not participating in CDSMP) in the study design, which limited the ability to determine treatment effects from baseline to post-intervention. On a larger systems note, the frequent workshop cancelations due to inadequate numbers of participants limited recruitment opportunities for this study. Despite expanding the possible data collection area to the entire state and extending the time period for collection, the sample size remained small. This small convenience sample reflects real world data collection using community-based interventions for research purposes. There were no resources allocated to the study. This smaller sample also reflects ongoing national difficulties recruiting participants into the CDSMP workshops even though the workshops have well-demonstrated participant retention rates (30). In future studies, partnering with larger, funded studies or agencies across states is recommended to expand recruitment opportunities and enlarge sample size.

Those adults who agreed to participate in this study may have been different from others in CDSMP who did not choose to participate in this particular study but may have agreed to participate in other ongoing research such as a concurrent Medicare study. Ritter et al. (31) commented on this type of bias associated with soliciting consent for a separate Medicare study from participants in the *National Study of CDSMP*; the consenting process for the Medicare study that was added produced a group of participants who were different from the main group in the main study in terms of number of conditions, use of healthcare visits, and even ethnicity.

Self-efficacy is understood as a dynamic construct that may change at any time (16), and CDSMP research generally has measured changes in SEMCD over a 6-month post period (18). This current research collected SE scale data generally at the last session rather than at 6 months, which may have produced different results from the 6-month post measures associated with other CDSMP research. The data collection associated with this study did provide a real-time snapshot of changes following engagement in CDSMP workshops that had not typically been presented in other research.

Given these exploratory results, additional research would be needed to clarify results further. SE (SEMCD or Fall-related SE) is an important component of health promotion programs, such as CDSMP and fall prevention programs. Higher SE facilitates health outcomes and self-management of conditions (18) and falls (7). Future studies should consider collecting baseline and post-participation scale data for both SE scales (SECMD scale and FallE Scale) across both CDSMP and fall prevention programs. This would facilitate comparison between groups taking these types of EBP and measure program potential effect on types of SE. While the FallE Scale was selected for use in this study, a variety of other fall-related SE scales exist. Future studies are encouraged to use this scale and/or other scales to document the robustness of the relationship between falls SE after CDSMP participation. Researchers should also explore if the shared content contained in fall prevention programing, such as action planning and emphasis on building mastery to manage falls can affect SEMCD. This could lead to more effective bundling and packaging of services for older adults.

CDSMP, as an EBP, not only facilitates building skills and SEMCD but also specifically addresses fall prevention via a recently added short segment to the standardized manual (11). Although aging adults commonly face increasing risk for both chronic disease (1, 32) and falls (2) with disease-related problems increasing risk of falls especially in women (3), the relationship between Fall-related SE and SEMCD had not been explored until now. This exploratory research highlighted a relationship between SEMCD and Fall-related SE even before workshop participation. Given the preliminary results showing changes in Fall-related SE post-participation in CDSMP, researchers may wish to consider exploring a broadened use of CDSMP as an early approach in fall prevention. Currently, the recommended EBPs for older adults include both fall prevention programs and disease self-management programs, such as CDSMP (4). This research takes an exploratory step toward Beattie’s recommendation (21) of an “inclusive approach to the effective management of chronic disease and the reduction of fall risk; an approach that values and enfolds the broad spectrum of healthy aging program offerings” (p. 62).

## Author Contributions

KG, MS, JH, KE, and MW meet the criteria for authorship in that they have all contributed to the design and analysis of the research in addition to providing ongoing comments and revisions during draft process. Each has reviewed the final draft submitted for publication and agreed to be held jointly accountable for the content and accuracy.

## Conflict of Interest Statement

There were no commercial or financial relationships that might be interpreted as a conflict of interest for authors during this research endeavor. This research was initially published as a manuscript chapter in dissertation entitled, *An exploration of self-efficacy among older adult participants in a disease self-management program*.
